# Safety and feasibility of reduced-port robotic distal gastrectomy for gastric cancer: a phase I/II clinical trial using the da Vinci Single Port(SP) robotic system

**DOI:** 10.1038/s41598-023-45655-6

**Published:** 2023-10-30

**Authors:** Sung Hyun Park, Youn Nam Kim, Jawon Hwang, Ki-Yoon Kim, Minah Cho, Yoo Min Kim, Woo Jin Hyung, Hyoung-Il Kim

**Affiliations:** 1 Department of Surgery, Yonsei University College of Medicine, 50-1 Yonsei-Ro, Seodaemun-Gu, Seoul, 03722 Korea; 2 Gastric Cancer Center, Yonsei Cancer Center, Yonsei University Health System, Seoul, Korea; 3 Department of Biostatistics, Graduate School of Public Health, Yonsei University, Seoul, Korea

**Keywords:** Cancer therapy, Gastrointestinal cancer

## Abstract

Minimally invasive surgery reduces surgical trauma and the size and number of incisions. The da Vinci SP robotic surgical system was designed to overcome the technical demands of single-incision laparoscopic surgery. This study aimed to demonstrate the safety and feasibility of single-port (SP) robotic distal gastrectomy (SPRDG) for patients with gastric cancer using the da Vinci SP system (Intuitive Surgical Inc., Sunnyvale, CA, USA). This study was designed as a single-arm prospective phase I/II clinical trial of SPRDG (first posted date: 21/09/2021, NCT05051670; clinicaltrials.gov). SPRDG using the da Vinci SP system was performed on 19 patients with gastric cancer between December 2021 and October 2022. The primary outcome was the safety of SPRDG as measured by major postoperative complications. The secondary outcomes were operation time, bleeding amount, bowel motility recovery, and length of hospital stay. SPRDG was performed in all 19 patients without unexpected events, such as use of additional trocars or conversion to laparoscopic or open surgery. No major complications occurred postoperatively (0/19, 0.0%). The mean operation time was 218 min (range 164–286 min). The mean hospital stay duration was 3.2 days (range 2–4 days). This phase I/II clinical trial, performed by a single expert surgeon, demonstrated the safety and feasibility of SPRDG with the da Vinci SP system in selected patients with gastric cancer. SPRDG could be a reasonable alternative to conventional or reduced-port minimally invasive gastrectomy, as it has cosmetic advantages, early recovery, and safe discharge.

## Introduction

Laparoscopic surgery has become an essential surgical option to treat patients with gastric cancer over the past two decades^1–4^. Laparoscopic surgery gives better cosmesis and earlier recovery with less postoperative pain than open surgery techniques^5–7^, especially when fewer trocars are used^8–10^. Although surgeons can suffer the limitations of the laparoscopic instrument and the fulcrum effect technically demanding, reduced-port laparoscopic surgery has been developed to minimize the number of trocar insertions^11–13^.

Robotic surgery systems have been widely used to overcome the restrictions of laparoscopic surgery technology^14–17^. The robotic systems have also evolved to a single-site platform to provide a reduced-port approach^18–20^. The da Vinci single-port (SP) system (Intuitive Surgical Inc., Sunnyvale, CA, USA) was developed for a more straightforward reduced-port approach^21–26^.

The earlier da Vinci Si and Xi systems (Intuitive Surgical Inc.) also allowed reduced-port surgery using a single-site system. However, less experienced surgeons found the docking to be challenging, which limited the widespread uptake of these systems. Thus, the da Vinci SP system was developed as a single-port-based multichannel cannula that maintains the advantages of conventional robotic surgical systems, such as high-resolution three-dimensional images and wristed anti-tremor action. Therefore, da Vinci SP is a promising system that combines robotic surgery with a reduced-port approach.

This prospective study aimed to confirm the applicability of reduced-port distal gastrectomy using the da Vinci SP system in patients with gastric cancer. We used surgical outcomes and early complication rates to assess the safety and feasibility of da Vinci SP robotic distal gastrectomy (SPRDG). Furthermore, we describe our initial experiences and surgical techniques to increase the potential application of the da Vinci SP robotic system.

## Methods

### Patients and study design

This clinical trial was a single-arm prospective phase I/II study conducted at a single institution, performed by a single surgeon with vast experience in both laparoscopic and robotic gastrectomy. We enrolled 19 patients with gastric cancer scheduled for robotic distal gastrectomy from December 2021 to October 2022. The first patient was enrolled on 16/12/2021 (SP-01). The inclusion criteria were a biopsy-confirmed adenocarcinoma of the stomach scheduled to undergo radical gastrectomy, age between 20 and 80 years, American Society of Anesthesiologists (ASA) physical status classification system class I–III, and signed informed consent after a thorough explanation of the study. The exclusion criteria were patients with distant metastasis, candidate for endoscopic mucosal resection, complicated gastric cancer (obstruction or perforation), another active primary tumor, and vulnerable subject (illiterate or pregnant). Patient characteristics, surgical factors, and pathology results of enrolled patients were prospectively collected. Informed consent was obtained from all patients after providing sufficient information about gastrectomy using the da Vinci SP robotic system. This phase I/II single-arm study was approved by the Institutional Review Board of Severance Hospital, Yonsei University Health System (4-2021-0881) and was conducted in compliance with the ethical guidelines of the Helsinki Declaration of 1975. The study protocol was registered at clinicaltrials.gov (first posted date: 21/09/2021, NCT05051670). All methods were performed in accordance with the relevant guidelines and regulations.

### Da Vinci SP system

Like the previous da Vinci Xi system, the da Vinci SP system consists of the surgeon console, patient cart, and vision cart^21,27–29^. The da Vinci SP system enables multiple flexible instruments to be docked in a single port, permitting surgeries to be conducted through smaller incisions with fewer ports. Additionally, four multiarticulated instruments and a three-dimensional camera can be inserted through a 25 mm multichannel cannula. The da Vinci SP system provides high-resolution three-dimensional surgical views and sophisticated full-wristed movements that minimize tremors, similar to the da Vinci Xi system. In addition, the newly added joint in the da Vinci SP system instrument acts like an elbow to enhance surgical access within the narrow multichannel cannula, thereby providing a broader working space for surgeons. Using the SP approach, surgeons can manipulate the multiarticulated instruments with a similar sense of control as that of conventional robotic surgery.

### Settings and preparation for da Vinci SP robotic distal gastrectomy

**Figure 1:**
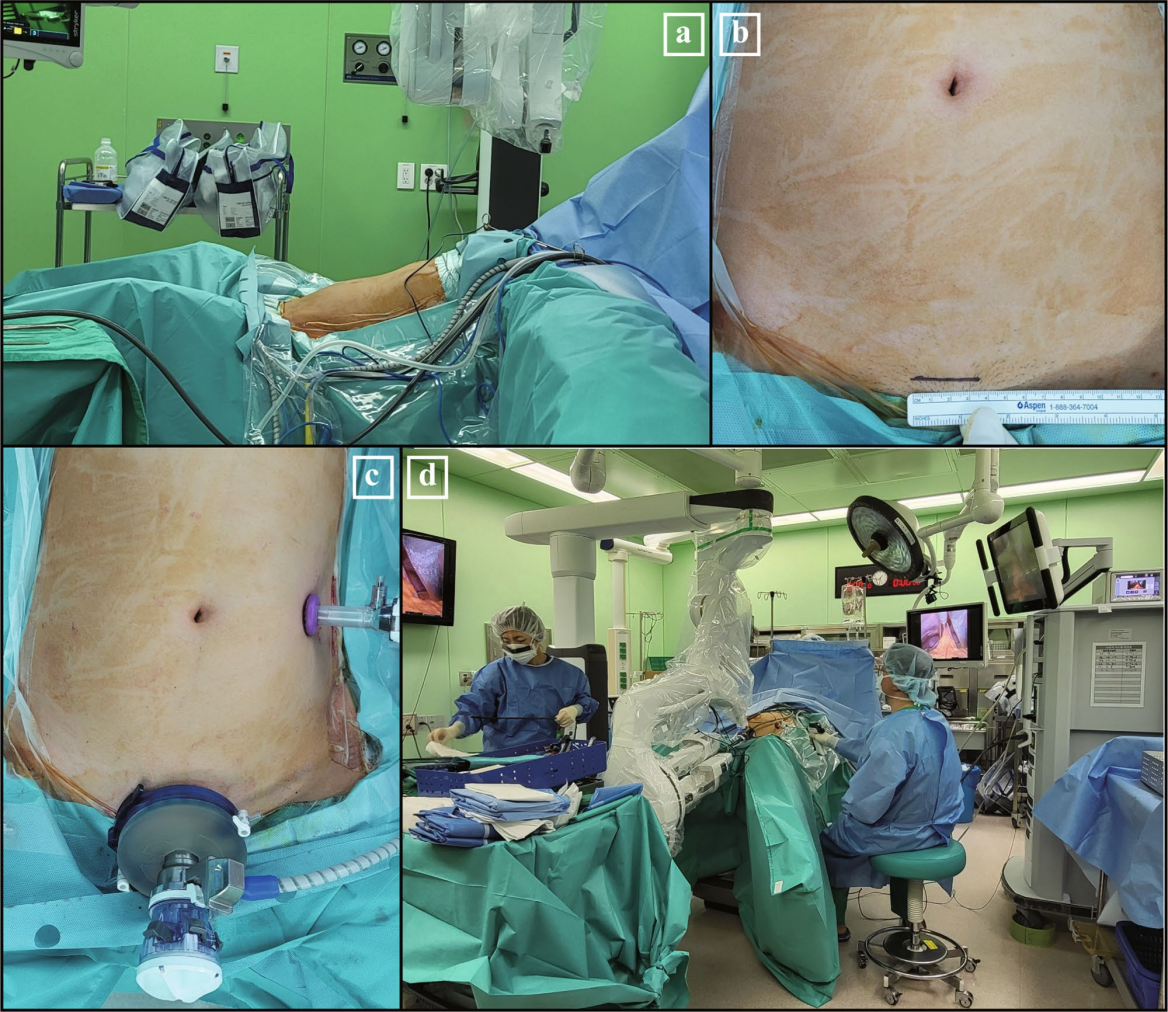
Setting up for da Vinci SP robotic distal gastrectomy (SPRDG). (**a**) Patient in lithotomy and 15-degree reverse Trendelenburg position; (**b**) Pfannenstiel incision for (**c**) multichannel cannula docking and multichannel cannular insertion, with additional assist trocar placement also shown; (**d**) patient-side preparation after the completion of docking for SPRDG.

Supplementary Figure S1 shows a schematic illustration of the instrument setup. Figure 1 demonstrates the preparation process for patient positioning and robotic system docking for SPRDG. The patient was placed in the lithotomy position for SPRDG (Fig. 1a). A Pfannenstiel incision of about 3.5 cm in length was made to create a pathway for the multichannel cannula instead of the conventional periumbilical midline incision (Fig. 1b). After pneumoperitoneum formation, a 15-degree reverse Trendelenburg position was set, and one additional 12 mm assist trocar was inserted into the patient’s right or left lower quadrant, depending on the reconstruction plan (Fig. 1c). Afterward, the patient cart from the caudal side and the multichannel cannula were docked to the da Vinci SP robotic system. A camera and three full-jointed instruments (arm 1: scissors capable of monopolar electrocauterization; arm 2: a Cadiere atraumatic forcep; and arm 3: Maryland forceps capable of bipolar electrocauterization) were docked through the single-port da Vinci SP cannula (Part number 430004, Part number 430010, and Part number 430009, respectively; Intuitive Surgical Inc.) and used during surgery. The additional assist trocar was used to transfer gauze and needles and to facilitate using the ultrasonic device Sonicision (Medtronic, Minneapolis, MN, USA) for tissue dissection during the surgery; linear stapling for anastomosis or transection using Signia (Medtronic); and vessel clipping. Figure 1d shows the initial landscape of the patient side view after the docking is complete.

### Surgical procedure

**Figure 2:**
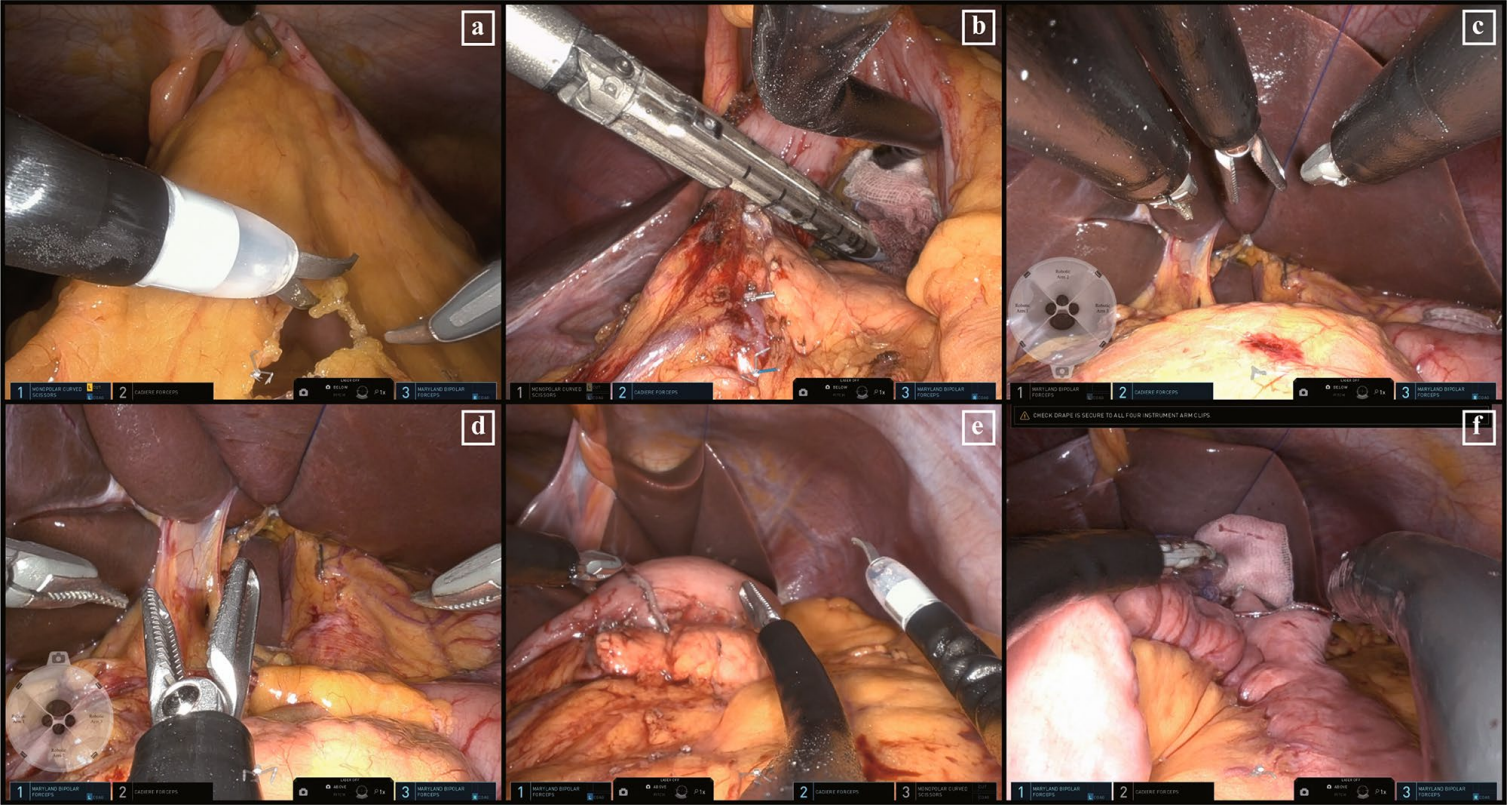
Intraoperative surgical view of SPRDG. (**a**) Instrument positioning, tissue traction, and dissection; (**b**) a linear stapler for duodenal transection through the assist trocar; (**c**) camera placement positioned below till the completion of station #6; (**d**) after duodenal transection, all instruments were rotated 180 degrees and the camera was positioned above for suprapancreatic lymph node dissection; (**e**) intracorporeal delta-shaped gastroduodenostomy stapled via trocars located on the patient’s left side; (2f.) gastrojejunostomy stapled via trocars located on the patient’s right side.

**Figure 3:**
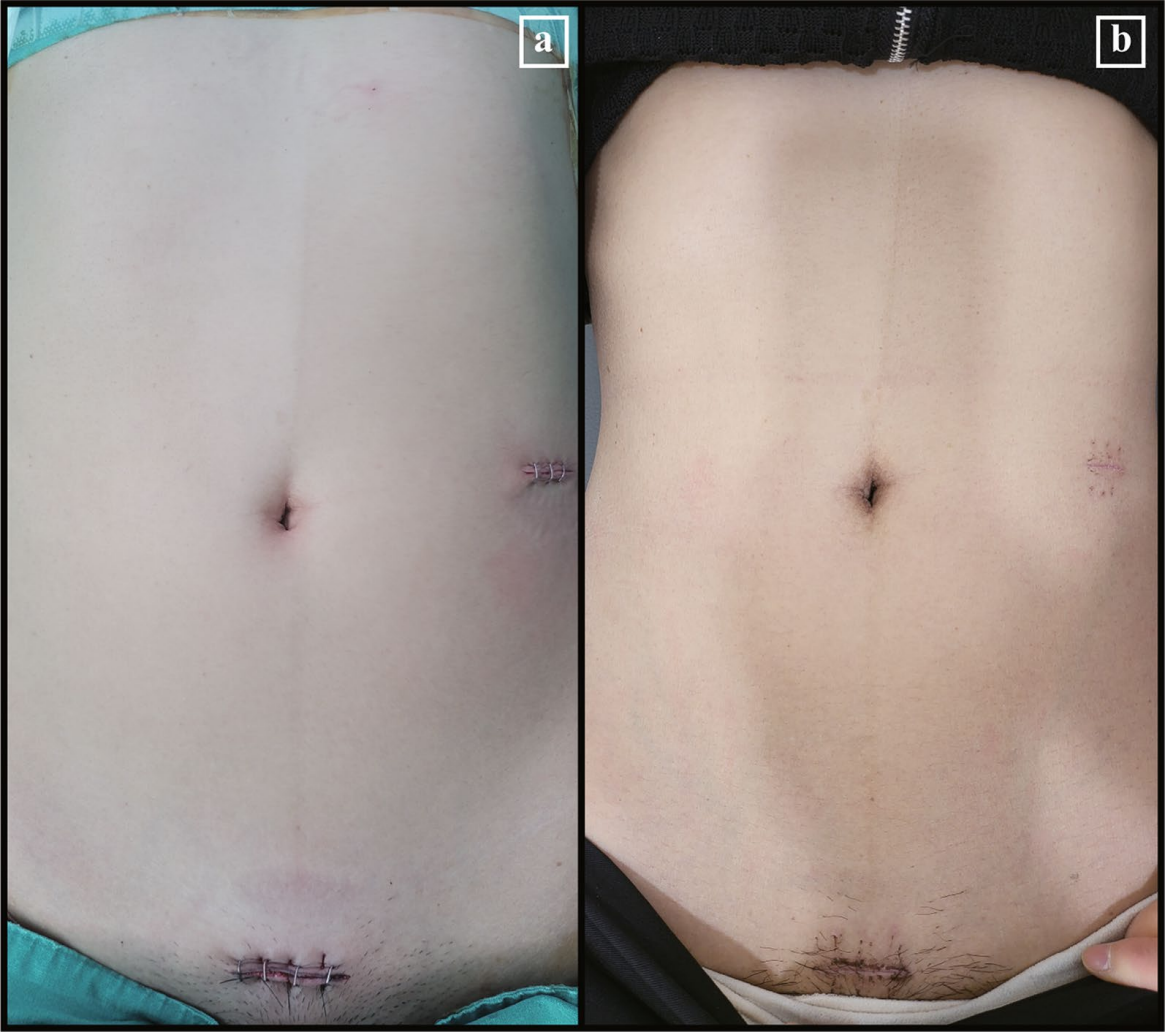
Postoperative wound after SPRDG. (**a**) Immediate postoperative wound; (**b**) 2-weeks postoperative wound after the wound-healing process.

All surgical procedures followed the gastric cancer surgery guidelines^3,30^. According to the Korean Practice Guideline for Gastric Cancer, D1 + lymphadenectomy was performed for early gastric cancer patients with any suspicion of LN metastasis, and D2 lymphadenectomy was performed for patients with suspicion of LN metastasis or advanced gastric cancer^3^. Dissection of the lymph nodes was performed while retracting tissue using Cadiere forceps in arm 2. The scissors in arm 1 and Maryland forceps in arm 3 were used for lymph node dissection (Fig. 2a). The duodenum was transected using a surgical stapler through the assist port (Fig. 2b). The cannula was rotated as needed to visualize the suprapancreatic area (Fig. 2c,d). After liver traction, suprapancreatic area lymph node dissection was performed using monopolar scissors and Maryland forceps. Then, the stomach resection was performed using a linear stapler. The stomach specimen was retrieved through the primary Pfannenstiel incision. After confirming the tumor location and margin length, reconstruction by intracorporeal delta-shaped gastroduodenostomy (Fig. 2e) or gastrojejunostomy (Fig. 2f) was performed as previously described^31^. The closure of the Pfannenstiel incision commenced with peritoneal closure using an absorbable purse-string suture. The rectus muscle was approximated with no suturing. The transversalis fascia was closed using barbed suture material with a continuous suture technique. Finally, the skin was approximated with vertical mattress sutures with a skin stapler (Fig. 3a).

### Postoperative management

All 19 patients who underwent SPRDG were treated with the same postoperative protocol as patients who undergo minimally invasive distal gastrectomy. In managing postoperative pain, every patient in this study was provided with IV PCA (Intravenous Patient-Controlled Analgesia). Additionally, a drainage tube was routinely placed in all patients after the procedure. According to the enhanced recovery after surgery protocol, patients were allowed sips of water from the evening of the operation day^32^. Regardless of gas passing, a liquid diet was followed from the morning of the first postoperative day, followed by a soft diet at noon and evening. If there were no specific complications until postoperative day 3, discharge was encouraged if the patient’s condition satisfied the previously reported safe discharge criteria^33^.

### Outcome measurement

The primary endpoint of this phase I/II SPRDG clinical trial was the 30-day major complication rate. Any further prescribed drugs, laboratory or image examinations performed on patients and treatment courses were reviewed. Any complications requiring outpatient clinic attendance, rehospitalization, or unexpected hospital visits within 30 days were observed. Whether complications developed or not was confirmed through a weekly quality control meeting as previously described^34^. Additionally, secondary endpoints of this single-arm study were operation time, bleeding amount, bowel motility recovery, and length of hospital stay.

### Statistical analysis

According to a previous report of complications of robotic subtotal gastrectomy, we set the threshold margin of the complication rate to 15%^17,35^. A single-stage single-arm phase II clinical trial design was used to test the null hypothesis that the complication rate was more than or equal to 15% and the alternative hypothesis that the complication rate was less than or equal to 4%. The required sample size was estimated to be 19 cases based on a significance level of 0.2 and a power level of 0.7. If the count of verified complications was equal to or less than two, the null hypothesis may be rejected. The sample size was calculated using the R package clinfun (Clinical Trial Design and Data Analysis Functions; R Foundation for Statistical Computing, Vienna, Austria). Categorical variables are reported as numbers and percentages, and continuous variables are reported as means and ranges. We calculated the complication rate estimate and its 80% confidence interval. All analyses were conducted using R software (version 4.1.0; R Foundation for Statistical Computing).

### Ethical statement

Informed consent was obtained from all patients after providing sufficient information about gastrectomy using the da Vinci SP robotic system. This phase I/II single-arm study was approved by the Institutional Review Board of Severance Hospital, Yonsei University Health System (4-2021-0881). The study protocol was registered at clinicaltrials.gov (NCT05051670).

## Results

The basic characteristics of the 19 patients who participated in the clinical trial are given in Table 1. The mean age of the patients was 54.7 years (range 35–70 years), and nine (47.4%, 9/19) were male. The entire surgical process of SPRDG using the da Vinci SP system was feasible and was successfully performed in all 19 patients. There were no technical problems with instrument docking or movement. No patient needed conversion to laparoscopic or open surgery or additional trocar insertion other than the initially planned assist port. Also, D2 lymph node dissection was successfully performed in three patients. Six patients underwent delta-shaped Billroth I gastroduodenostomy, and the other thirteen patients underwent Billroth II gastrojejunostomy.

**Table 1:** Demographics, tumor characteristics, and surgical features of da Vinci SP robotic distal gastrectomy patients.

Characteristics	Robotic SP distal gastrectomy (n = 19)
Age, mean (range), years	54.7 (35.0–70.0)
Sex, n (%)	
Male	9 (47.4)
Female	10 (52.6)
BMI, mean (range), kg/m^2^	24.4 (19.7–27.8)
ASA score, n (%)	
1	2 (10.5)
2	13 (84.2)
3	1 (5.3)
Previous abdominal surgery, n (%)	
Yes	5 (26.3)
No	14 (73.7)
Tubular location, n (%)	
Middle third	10 (52.6)
Lower third	9 (47.4)
Circular location, n (%)	
Lesser curvature	4 (21.1)
Greater curvature	8 (42.1)
Anterior/posterior wall	7 (36.8)
cT, n (%)	
cT1	16 (84.2)
cT2–3	3 (15.8)
cT4a	0 (0.0)
cN, n (%)	
cN0	19 (100.0)
cN +	0 (0.0)
Resection, n (%)	
Distal gastrectomy	19 (100.0)
Dissection, n (%)	
D1 +	16 (84.2)
D2	3 (15.8)
Reconstruction, n (%)	
Billroth I	6 (31.6)
Billroth II	13 (68.4)

*SP* single port; *BMI* body mass index; *ASA* American Society of Anesthesiologists; *cT* clinical tumor stage; *cN* clinical nodal stage.

### Perioperative outcome

Table 2 summarizes the outcome of SPRDG in 19 patients. In all 19 patients, a soft diet was possible at maximum 2 days after surgery, and gas passing was confirmed as evidence of bowel movement recovery after a mean of 2.1 days (range 1–3 days). According to the discharge criteria protocol^33^, all patients were discharged after a mean of 3.2 days (range 2–4 days) of postoperative hospital stay. There were no early postoperative major complications of Clavien-Dindo classification grade III or higher. The major complication rate was 0% (0/19), and the overall complication rate was 26.3% (5/19). Among the reported postoperative complications, two patients experienced postoperative fever and were treated with either antipyretics or antibiotics. Two others faced postoperative urinary retention. Chylous ascites developed in one patient treated with a low-fat diet and orlistat, but no rehospitalization or unscheduled hospital visit occurred on follow-up to 30 days postsurgery.

**Table 2:** Postoperative outcomes after da Vinci SP robotic distal gastrectomy.

Characteristics	Robotic SP distal gastrectomy (n = 19)
Operation time, mean (range), minutes	218 (164–286)
Bleeding amount, mean (range), mL	50 (9–150)
Conversion to laparoscopy or open, n (%)	
Yes	0 (0.0)
No	19 (100.0)
Tumor size, mean (range), mm	24.3 (10.0–72.0)
R0 resection	
Yes	19 (100.0)
No	0 (0.0)
Number of retrieved LNs, mean (range), n	29.7 (16.0–48.0)
Stage (AJCC 8th), n (%)	
I	17 (89.5)
II	2 (10.5)
III	0 (0.0)
Diet buildup, mean (range), days	
Sips of water	0.7 (0–1)
Liquid diet	1.1 (1–2)
Soft diet	1.2 (1–2)
Gas passing	2.1 (1–3)
Length of hospital stay, mean (range), days	3.2 (2–4)
^a^Complications, n (%)	
No	14 (73.7)
Grade I	2 (10.5)
Grade II	3 (15.8)
Grade III or higher	0 (0.0)
^a^Readmission	
Yes	0 (0.0)
No	19 (100.0)

*SP* single port; *LN* lymph node; *AJCC 8th* The Eighth Edition American Joint Committee on Cancer Cancer Staging.

^a^30-day complication rate or readmission rate, according to the Clavien-Dindo classification system.

### The postoperative wound after SPRDG

The Pfannenstiel incision was used to maximize the advantage of a wide working space from small incisions that the da Vinci SP system offers, instead of the transumbilical incision used in conventional laparoscopic or robotic gastrectomy. The da Vinci SP system’s multiarticulated instrument and camera view allowed distal gastrectomy to be conducted through the Pfannenstiel incision without approach or vision problems. A representative example of an immediate postoperative wound is shown in Fig. 3a, and the wound 2 weeks after the healing process is shown in Fig. 3b. From a cosmesis standpoint, only a minimal wound that can be hidden by pubic hair or underwear remained.

## Discussion

In this study, we reported da Vinci SPRDG could be safely performed without major complications in patients with gastric cancer. All 19 patients were discharged within 4 days after surgery. Among them, fourteen patients completed their hospital stay within 3 days, and no patients had an unscheduled hospital visit within 30 days postsurgery.

**Figure 4:**
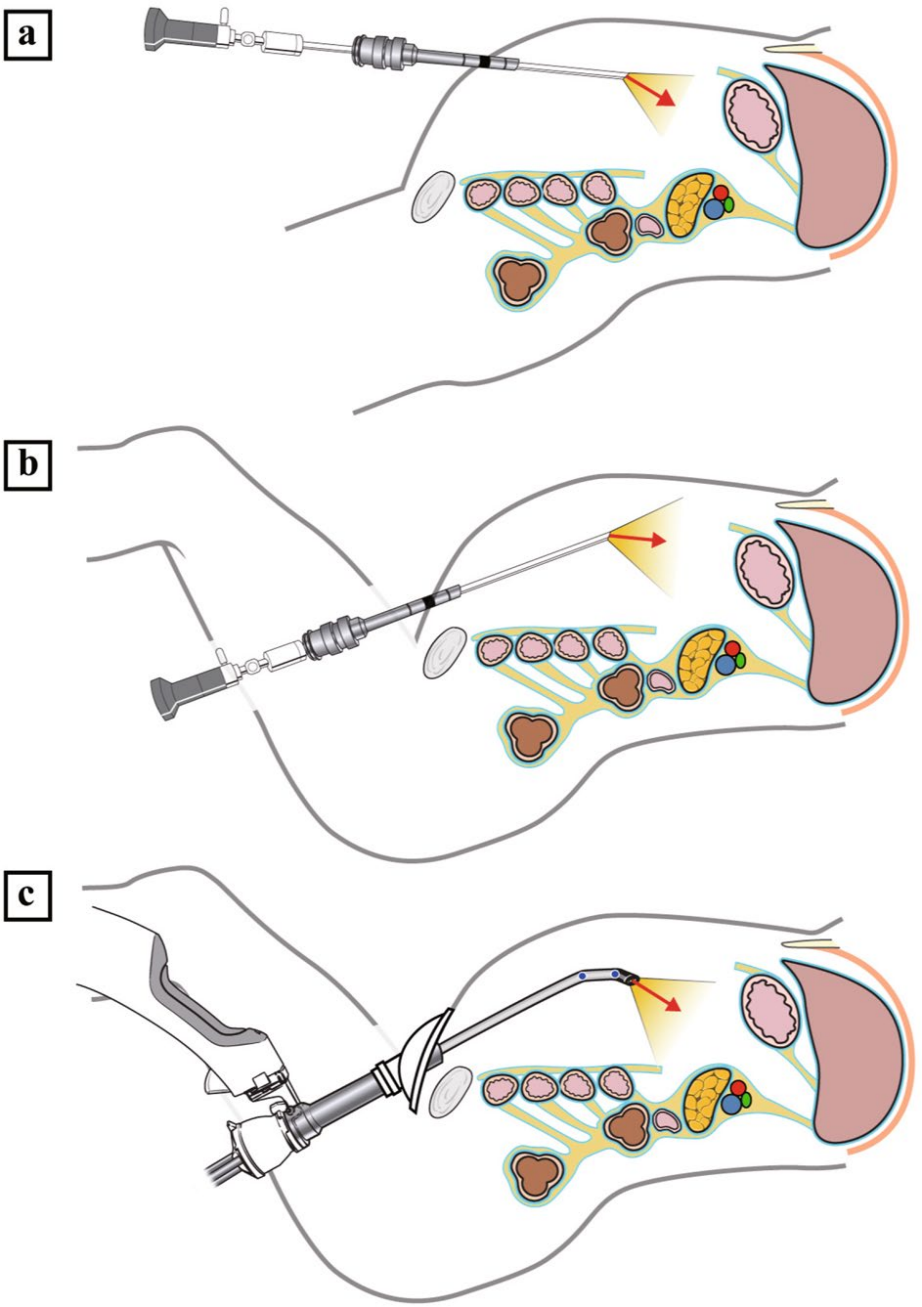
Schematic illustration of robotic gastrectomy. (**a**) Camera view range of conventional robotic gastrectomy or laparoscopic gastrectomy through a periumbilical midline incision with a rigid 30-degree camera; (**b**) imaginary camera view range through a Pfannenstiel incision with a rigid 30-degree scope; (**c**) camera view range of da Vinci SP robotic gastrectomy through a Pfannenstiel incision with an articulated camera.

We selected the Pfannenstiel incision for multichannel cannula insertion and specimen retrieval. Classically, the Pfannenstiel incision is favored for colorectal and gynecologic surgery because it has lower incisional hernia incidence and less postoperative pain^36–43^. For gastric cancer surgery, surgeons usually prefer a periumbilical midline incision (Fig. 4a). In conventional laparoscopic or robotic gastrectomy, including the da Vinci Xi system, a rigid robotic camera usually cannot provide an appropriate surgical view through a Pfannenstiel incision (Fig. 4b). The exposure of an accurate surgical field with anatomic landmarks is difficult in conventional robotic gastrectomy, especially when dissecting the suprapancreatic area.

The camera of the da Vinci SP robotic system is multijointed and includes an elbow joint, so that the surgical area in the upper abdomen is sufficiently accessed and visualized through the Pfannenstiel approach (Fig. 4c). Herein, in this prospective study, a Pfannenstiel approach in the lithotomy position was chosen for use with the new technology of the da Vinci SP system. The advantages of a Pfannenstiel incision over conventional techniques, including lower incisional hernia rate, less postoperative pain, and better cosmetic effect, are expected to maximize the benefits of this minimally invasive surgery.

Since the introduction of minimally invasive surgery, surgeons have been trying to perform surgically and oncologically safe operations with less trauma. Instruments and imaging systems have been developed to enable comparable or even better dissection quality than conventional surgery with a smaller incision. If surgery can be conducted safely with fewer trocars, then the use of fewer trocars would reduce surgical trauma and consequent adverse effects. Indeed, this phase I/II clinical trial demonstrated that SPRDG through a Pfannenstiel incision using the da Vinci SP system could be a feasible and safe option for gastric cancer surgery.

Single-incision surgery and natural orifice transluminal endoscopic surgery are attractive not only because of lower surgical trauma but also because of reduced scarring. Of course, surgical and oncological safety must first be established. So, if the safety of the operation is qualified, the cosmetic satisfaction from small scars could be valuable for selective patients. This study’s patients had low transverse abdominal wounds that can be covered by underwear. Thus, SPRDG is expected to be a safe surgical option for patients for whom cosmesis is an important factor.

To the best of our knowledge, this study is the first report on the clinical experience of the da Vinci SP system with SPRDG. There was no conversion to other surgical techniques, additional trocar insertion, or grade III or higher major postoperative complications or readmission events in the 19 patients, demonstrating the feasibility and safety of the SP robotic system for distal gastrectomy. However, the use of the Pfannenstiel incision might affect early recovery and good cosmesis. Our procedure could be a valid option for selected patients with the potential for less postoperative pain, lower possibility of incisional hernia, and better cosmesis than standard surgical techniques.

There are several limitations to this study. First, as this was a single-arm study, we could not compare SPRDG with conventional robotic distal gastrectomy or laparoscopic distal gastrectomy, so comparative studies should be conducted to determine if SPRDG has advantages in terms of incisional hernia incidence, postoperative pain or cosmesis satisfaction. Second, we did not assess long-term patient outcomes in this study, which will be important to measure, considering that the most critical factor in radical cancer surgery is oncological safety. Third, although our study was prospective, the primary purpose was to confirm the safety of SPRDG, so there might be selection bias. Additional real-world studies may be needed in patients with challenging conditions such as high body mass index or advanced gastric cancer. Additionally, this study was conducted by a single expert surgeon with various experiences in laparoscopic and robotic gastrectomy. Whether this is generally accepted requires further research. Last, SPRDG procedure described in this manuscript was developed for initial cases, and methods are continuously evolving and undergoing modifications. For instance, we recently used bipolar dissectors in both hands rather than monopolar scissors in the left hand and a bipolar dissector in the right hand. The pursuit of optimizing surgical techniques is ongoing, and the surgical details discussed here may change with increased use of SPRDG.

## Conclusion

This phase I/II clinical trial, performed by a single expert surgeon, demonstrated the safety and feasibility of SPRDG in selected patients. SPRDG could be a reasonable alternative to conventional or reduced-port minimally invasive gastrectomy, with cosmetic advantages, early recovery, and safe discharge.

### Supplementary Information

Supplementary Figure S1.

## Data Availability

Hyoung-Il Kim had full access to all data in the study and took responsibility for the integrity of the data and the accuracy of data analysis.
