# Supplementary material for: Safety and feasibility of reduced-port robotic distal gastrectomy for gastric cancer: a phase I/II clinical trial using the da Vinci Single Port(SP) robotic system
Source: Sci Rep. 2023 Oct 30;13:18578. doi: 10.1038/s41598-023-45655-6 (PMC10616185; doi:10.1038/s41598-023-45655-6)
Supplement: Supplementary file 1 — Supplementary Figure S1. [file 41598_2023_45655_MOESM1_ESM.docx]

**Supplementary Material**

**Supplementary Fig. S1.** Schematic illustration of single-port robotic distal gastrectomy.

**
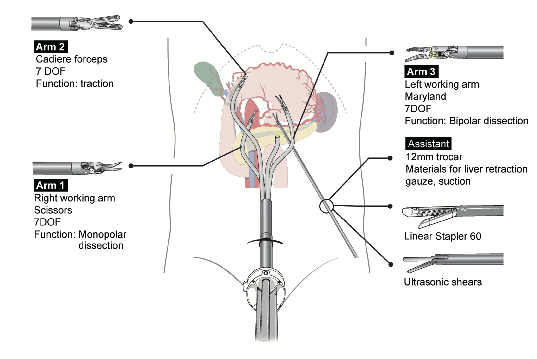
**
